# Water Availability–Demand Balance under Climate Change Scenarios in an Overpopulated Region of Mexico

**DOI:** 10.3390/ijerph18041846

**Published:** 2021-02-14

**Authors:** Jessica Bravo-Cadena, Numa P. Pavón, Patricia Balvanera, Gerardo Sánchez-Rojas, Ramón Razo-Zarate

**Affiliations:** 1 Universidad Autónoma del Estado de Hidalgo, Área Académica de Biología, Carretera Pachuca-Tulancingo s/n, Cd. Universitaria, Pachuca 42184, Mexico; gsanchez@uaeh.edu.mx; 2 Universidad Nacional Autónoma de México, Instituto de Investigaciones en Ecosistemas y Sustentabilidad, Antigua Carretera a Pátzcuaro # 8701, Ex-Hacienda de San José de la Huerta, Morelia 58190, Mexico; pbalvanera@cieco.unam.mx; 3 Universidad Autónoma del Estado de Hidalgo, Área Académica de Ciencias Agrícolas y Forestales, Av. Universidad Km.1, Ex hacienda de Aquetzalpa, Tulancingo 42000, Mexico; rrazo29@yahoo.com.mx

**Keywords:** climate change, mapping water availability, Sierra Madre Oriental, WaterWorld software

## Abstract

Climate change scenarios show that water availability could be decreasing in the near future, adding to the increasing problem of the growing water demands in socioeconomic sectors. The aim of this work was to generate a geographically explicit water balance concerning availability vs. demand in an overpopulated region of Mexico. Water balance and water deficit models were made for three periods of time: 1970–2000, and two future periods of time (2041–2060 and 2061–2080). Three global climate models were used in addition to two different climate scenarios from each (Representative Concentration Pathways (RCP) RCP 4.6 and RCP 8.5). Water demand for socioeconomic sectors was calculated through the water footprint. Water availability was 197,644.58 hm^3^/year, while that the water demand was 59,187 hm^3^/year. The socioeconomic sectors with the highest demand were domestic services (48%), agriculture (27%), livestock agriculture (20%), and timber production (5%). The highest water availability areas were not the same as those with the highest demand and vice versa. However, 39% of municipalities had a higher water demand than its availability. A significant reduction in water availability was identified, considering an interval of −15% to 40%. This variation depends on climate models, scenarios, and period of time. Areas with overpopulated cities in the region would have higher pressure on water availability. These results could be used in the implementation of public policies by focusing on adaptation strategies to reduce water deficit in the immediate future.

## 1. Introduction

Water supply is a fundamental element of the socio-environmental system (SES). However, due to climate change, SES has been heavily impacted because rainfall is changing in distribution, magnitude, and frequency, both globally and regionally [1,2]. In many regions, rainfall could decrease significantly. Meanwhile, water demand is increasing as a consequence of overpopulation and agricultural irrigation demand, as well as demand from other economic sectors. Therefore, a severe shortage of water might be expected, especially in arid countries with high overexploitation of their aquifers. Water demands in the agricultural sector, as part of the water–food nexus, will worsen problems caused by climate change. Agriculture has a huge global imprint through irrigation, and it could become the sector with the highest water demand, putting strong pressure on water resources worldwide [3]. On the other hand, it is estimated that there will be a 10–20% growth in the number of people at risk of hunger by 2050 [4]. Given this context, it is very important to assess the water availability–demand balance to estimate prospective water scarcity under future climate change scenarios.

An essential limitation for evaluating water demand on both the regional and local scales is the scarcity or lack of data in developing countries, mainly. Therefore, other parameters can be used as proxies for water demand. For example, in Mexico, one of these parameters is the advised use or volume of water authorized to public uses by the government [5,6]. This parameter refers to the volume of water concessions for a variety of public uses at the municipal and local levels. Another parameter that can be used as proxy for water demand is the water footprint. This refers to the amount of water used in the entire processing chain of goods and services produced for different sectors, such as agriculture and industry [7].

Worldwide water demand is about 4600 km^3^/year, and it is estimated that it will grow 30% by 2050 [5,6]. USA, China, and India represent, as a whole, 38% of the global water footprint [7]. Mexico’s per capita water consumption is above the international average at 1978 m^3^/year; this country is considered an importer of water [7]. Most water is used for irrigation (78%) and only 15% for domestic use [5,6]. Additionally, Mexico is not a self-sufficient country with regard to food, including corn, which is the staple food of the population. While the water demand for irrigation, industry, and public services is increasing, water availability is not owing to the deforestation, land degradation, and climate change [8]. Moreover, Mexico’s geographic characteristics increase its vulnerability to climate change due to higher sensibility to extreme weather events [6,9,10]. Under certain future climate change scenarios in most of the Mexican territory, the annual mean precipitation could be reduced by up to 10% [11]. Currently, the demand for water has led to 40% of Mexico’s aquifers being over-exploited [5,6]. As a consequence, an increase in water deficit would mean a risk to human welfare.

This research was focused on Mexico’s central-east region, which is one of the most overpopulated regions in the country with high influence on the megalopolis of Mexico City with 37 million inhabitants. As a consequence of overpopulation, there are strong pressures to the ecosystems for basic services such as the provision of water, food, among others. For this reason, generating basic information to design adaptation strategies and management of hydrological services is a vital task. In this study, the availability and demand of water in this large region of Mexico were analyzed in order to identify areas with water deficit currently and in the near future under scenarios of climate change. We assume an increase in the water deficit as a result of both the decrease in precipitation due to climate change and the demand for irrigation water. Considering the high population density, the water deficit scenario could generate drastic economic and social problems. This research analyzes the water availability and demand in several socioeconomic sectors under the influence of future climate scenarios for a large region of Mexico, and it is one of the first assessed with downscaling data. The results are important because they provide a basis to establish adaptation strategies that could reduce the vulnerability of the area, particularly in large cities.

## 2. Materials and Methods 

### 2.1. Study Area

**Figure 1:**
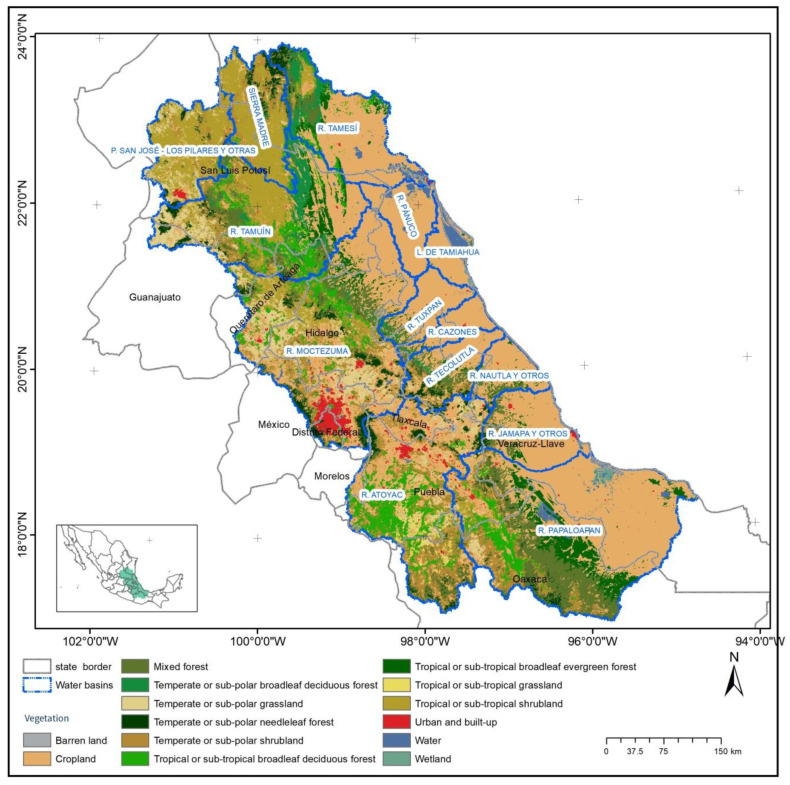
Location of the study region in central-eastern Mexico, covering five states of the country. It shows the land use in the region and the localization of the fourteen watersheds (R. = rivers).

The study area was the central-east region of Mexico, which has an extent of 199,830 km^2^. It includes 14 watersheds and 60 sub-watersheds that drain into the Gulf of Mexico (Figure 1). The area contains 589 municipalities (county equivalents) and 40,325 localities (towns), with 20,501,496 inhabitants, which represents 18.25% of the country’s total population. The Sierra Madre Oriental, a mountain belt that runs through the region from north to south, has a large influence on the climate and topography of the area. Due to the high environmental heterogeneity of the region, it contains a considerable variety of tropical and temperate vegetation types, as well as agroforestry systems, conventional crop agricultural systems and livestock [12] (Figure 1). About 42% of the study area is used for crop agriculture, 23% is scrubland, 11% is tropical forest, 8% mixed forest, 6% temperate forest, 6% used for livestock, and only 1% urban area [13].

### 2.2. Surface Water Availability

WaterWorld software was used to create spatially explicit hydrological surface models [14]. This program allows mapping on different scales, using global as well as regional databases [15,16]. The inputs variables to the models were rainfall, fog, ice, snow, and surface water flows and the outputs were evaporation, evapotranspiration by vegetation coverage, land use, and temperature [14,17]. Vegetation coverage is a very important factor because of its contribution to infiltration processes. For this work, the forest coverage was calculated using the data from high-resolution global maps of 21st-century forest cover change [18]. On the other hand, the temperature and rainfall data were obtained from WorldClim [15,19].

WaterWorld represents the hydric balance to the pixel level, showing water flows, rainfall, and fog (removing evaporation). Models were built with historical climate data from the period 1970–2000 (as a baseline or current scenario) and for the years 2060 and 2080 under climate change scenarios, these are based on a 1 km^2^ resolution per pixel. All results were processed in a geographic system in raster format using ArcGIS 9.3.

### 2.3. Water Demand

Water demand was estimated at the municipality level in the states of Queretaro, San Luis Potosí, Hidalgo, Veracruz, and Puebla, using estimates of water footprint in hm^3^/year (1 hm^3^ (cubic hectometer or MCM) = 1,000,000 m^3^). Water demand was calculated only with current data, future demand scenarios were not developed. The priority socioeconomic sectors such as domestic, crop agriculture, livestock, and forestry were considered. For the domestic sector, the number of inhabitants per locality [20] and water footprint values per inhabitant were used. For urban populations, a value of 1441 m^3^ annual per capita consumption (average for Mexico) and 1009 m^3^ for rural areas were used [5,7,21,22]. For the crop agricultural sector, the average annual production of crops (2000–2012 period) was used (Table 1) [23]. For livestock production, swine, cattle, sheep, and poultry were included, as well as beekeeping. Water footprint values per ton of each product were estimated using information from previous studies [5,7,22,24]. For timber production, the water footprint considered was between a range of 726 and 650 m^3^ water/m^3^ roundwood [25,26].

### 2.4. Climate Change Scenarios for 2060 and 2080

Different general circulation models (GCMs) were used, considering the Couple Model Intercomparison Phase 5 (CMIP5) scenarios. Three were selected: Geophysical Fluid Dynamics Laboratory GFDL-ESM2G (GFDL-CM3), Atmosphere and Ocean Research Institute, The University of Tokyo (MIROC5), and Community Climate System Model (CCSM4). These three GCMs have been used extensively and globally for climatic research of different topics, and they were used to evaluate hydrological risks in Mexico and other countries of America [11,27,28,29]. Moreover, two Representative Concentration Pathways (RCP 4.5 and 8.5) were used to perform climate change scenarios. RCP 4.5 is considered as an intermediate scenario. It predicts climate under the assumption that the present level of carbon dioxide emissions is maintained between 580 and 720 ppm, and it represents a global average warming ranging from 1.06 to 2.59 °C. The RCP 8.5 is the highest greenhouse gases emission scenario, it considers that the level of carbon dioxide emission changes drastically (>1000 ppm). It represents a global average warming ranging from 2.63 to 4.81 °C. Two periods of time were considered as follows: 2041–2060 and 2061–2080 [30,31,32].

A total of 12 hydrological projections for three GCM-CMIP5 model ensembles, two future climate projections, and two periods of time were analyzed in the study. We prefer using these three GCMs in order to be able to contrast the results with those obtained in other studies in Mexico. However, we are aware of that the results could have been different if we had used another GCM.

### 2.5. Water Deficit

Current and future water deficit were calculated as the difference between water demand and water availability by municipality using ArcGIS 9.3. For future deficit, the current demand was used, without making projections of demand growth due to population changes. For future water availability, only the 2061–2080 period was used, considering three GCM-CMIP5 climate models, and only one emission scenario, which is the RPC 8.5, represents the most extreme of all. Maps were used to show the spatial distribution of the water availability–demand balance. To support interpretations, water demand and water availability were mapped independently. The type of water source (surface or underground) for consumption use was also included.

## 3. Results

### 3.1. Surface Water Availability

**Figure 2:**
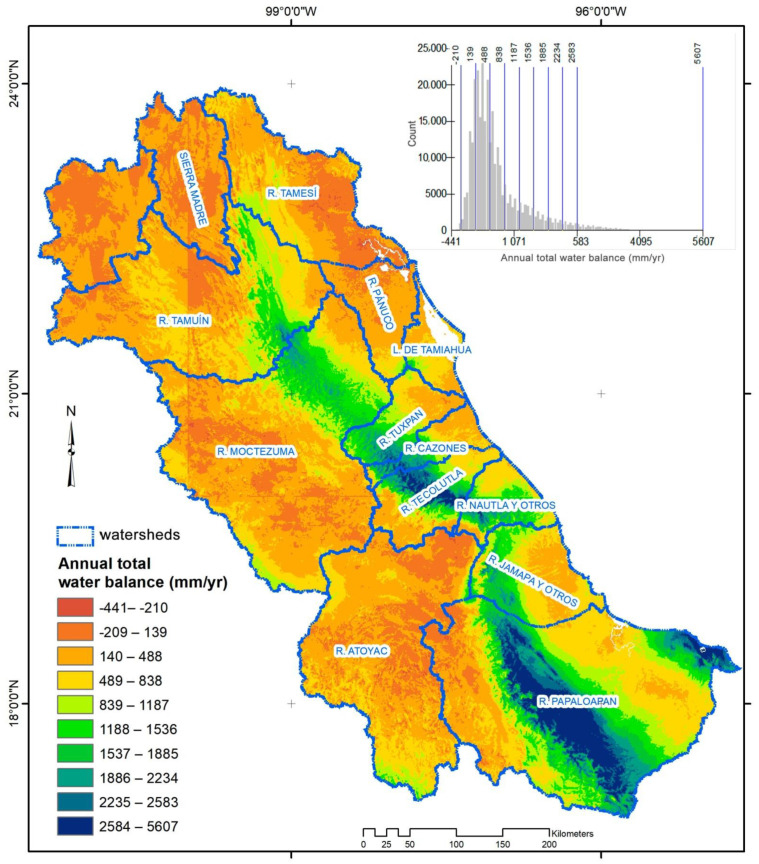
Current water availability surface in the central-eastern region of Mexico (unit: mm/year per pixel), and histogram of frequencies.

Water availability was 197,644,575 mm/year for all regions with an average of 663 mm/year (standard deviation = 698 mm/year) per watershed and range per pixel of −441 to 5607 mm/year. Fifty percent of the pixels had values between 0 and 500 mm/year, 15% had values higher than 2000 mm/year, and 5.5% of pixels had negative values indicating high water stress (Figure 2). Water availability was the highest value in the mountainous regions of the Sierra Madre Oriental (Figure 2) from the Papaloapan, Moctezuma, and Tamuin rivers watersheds. These watersheds cover 68% of the study area.

### 3.2. Climate Change Scenarios

**Figure 3:**
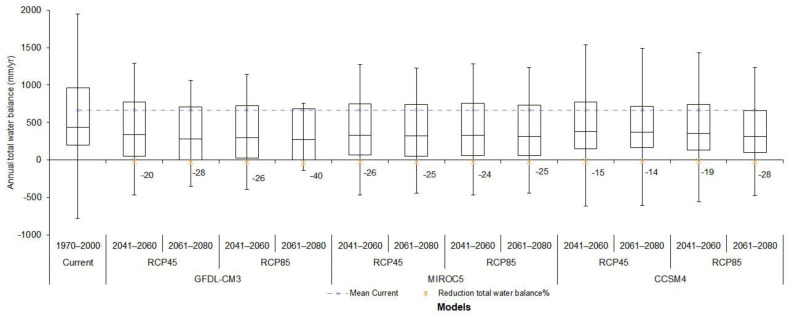
Boxplots of current and future water yield for three global circulation models (GCM-CMIP5) climate models and two under emission scenarios Representative Concentration Pathways (RCP) 4.5 and RCP 8.5. The dotted line indicates the mean of the present annual total water balance. The upper and lower bounds represent the 25th and 75th percentiles, respectively. The solid horizontal black line within the box corresponds to the median. The negative number represents the percentage reduction of total water balance in each model.

The surface water balance was different for the three GCM-CMIP5 model ensembles, two RCP future climate projections, and two periods of time. Results from surface water balance suggest that the future mean and median of two emission scenarios would be lower than that at the current time for the three climate models. Boxplots of present and future simulated annual total water balance for the GCM-CMIP5 model ensembles, scenarios, and the periods are presented in Figure 3. In general, the model that predicts a greater reduction in total water availability was GFDL-CM3 (20–40%), contrasting with the CCSM4 that predicts a lower reduction (15–28%). As expected, on RCP 4.5 scenario, water availability was reduced by between 15% and 26% by 2050 and 14–29% by 2080, whereas for RCP 8.5, the reduction was greater; 19–26% by 2050 and 25–40% by 2080 (Figure 3).

**Figure 4:**
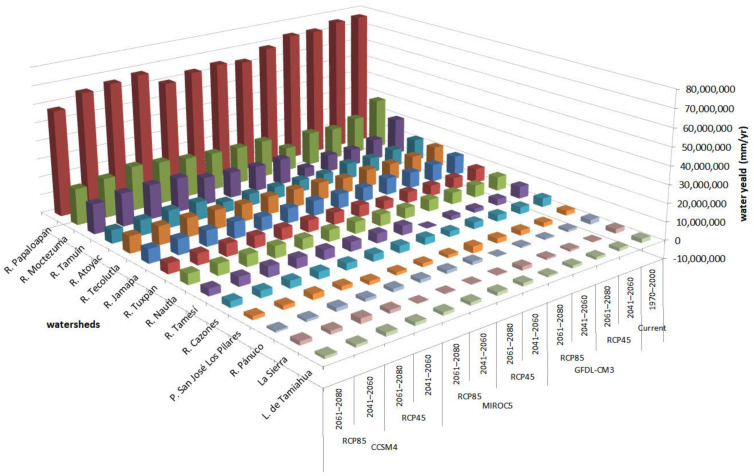
Surface water availability by watersheds for the present and the future (2060 and 2080) under two scenarios of climate change.

**Figure 5:**
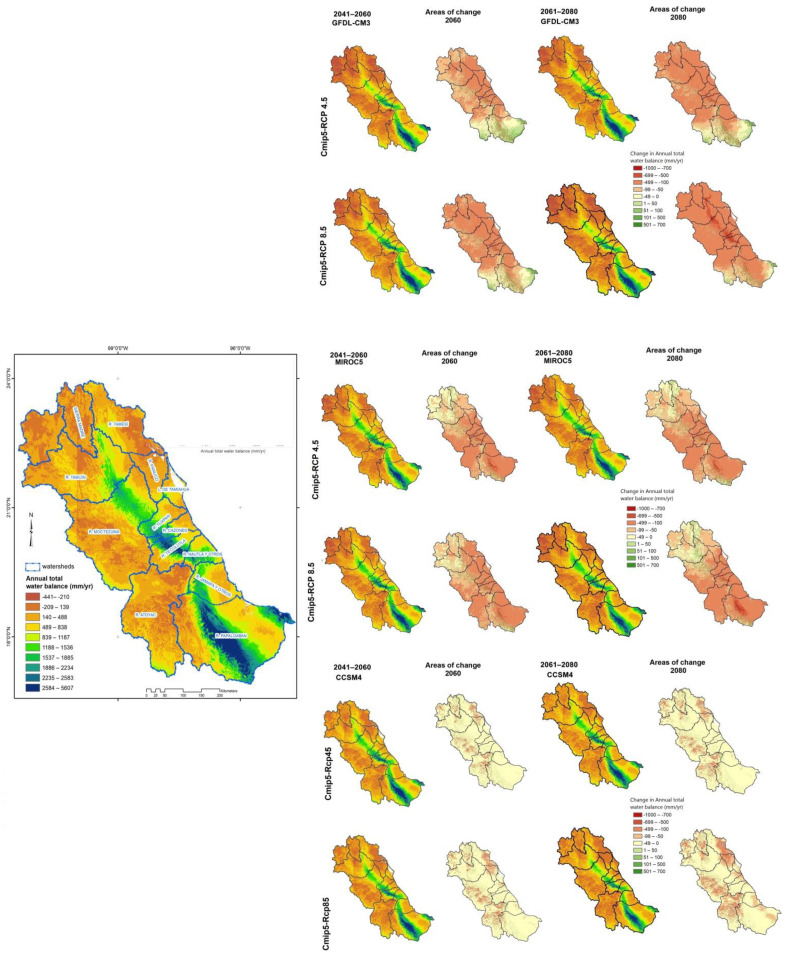
Comparison of surface water availability and change, between GCM-CMIP5 climate models under two emission scenarios (RCP 4.5 and RCP 8.5). That is the displayed result of the present water balance and spatial change in annual total water balance (mm/year) between the present and future scenario (2041–2060 and 2061–2080).

For 2050, spatial trends in the two RCP scenarios were similar but different when comparing between GCM-CMIP5 models, identifying severe changes in the north and southeast study area. For 2080, the trend slightly intensifies in the Panuco, Tamiahua, Moctezuma, and Atoyac river watersheds. Even though only 14% of the region had negative availability values, 56% of the area had values between 0 and 500 mm/year and only 11% had values above 2000 mm/year (Figure 4). In RCP 8.5, the reduction in water availability was greater in the lower part of the Panuco and Tamiahua river watersheds in the northeast of the study area with up to −500 (mm/year per pixel) change in annual total water balance. The same result was also observed in the Moctezuma and Atoyacán river watersheds (Figure 4 and Figure 5).

P. San José-Los Pilares and La Sierra are two watersheds with special interest because in both the reduction of water availability, with respect to current scenario, was found to be the highest with −150% and −117%, respectively. These water availability reductions occurred in all scenarios of GFDL-CM3 and MIROC5 models (Figure 4).

### 3.3. Water Demand

**Figure 6:**
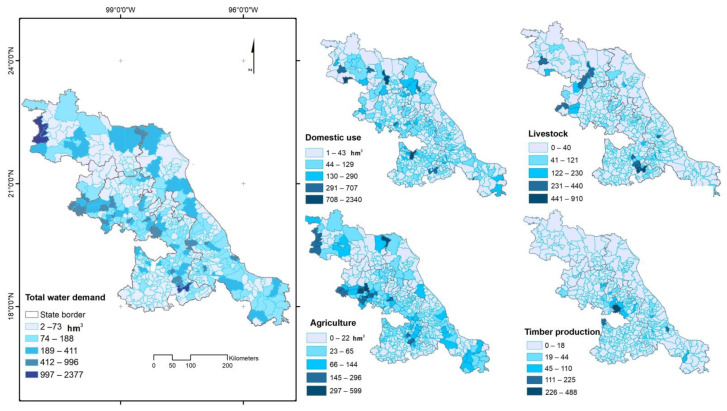
Geographic distribution of the total water demand and disaggregated by socioeconomic sector, current estimation.

The greatest demand was found in association with domestic use (48%) followed by crop agriculture (27%), livestock agriculture (20%), and timber production (5%). Total water demand was 59,187 hm/year with an average of 49 hm^3^/year per municipality (118 hm^3^/year) and a range of 1.5 to 2377 hm^3^/year (Figure 6). Contrarily, the cities with the highest water demands were Puebla (2377 hm^3^/year), San Luis Potosi (1594 hm^3^/year), and Queretaro (1570 hm^3^/year). In the crop agricultural sector, the highest values ranged from 431.1 to 599.1 hm^3^/year. In the forest sector, only two municipalities accounted for 24% of the water demand (Figure 6).

### 3.4. Water Deficit

**Figure 7:**
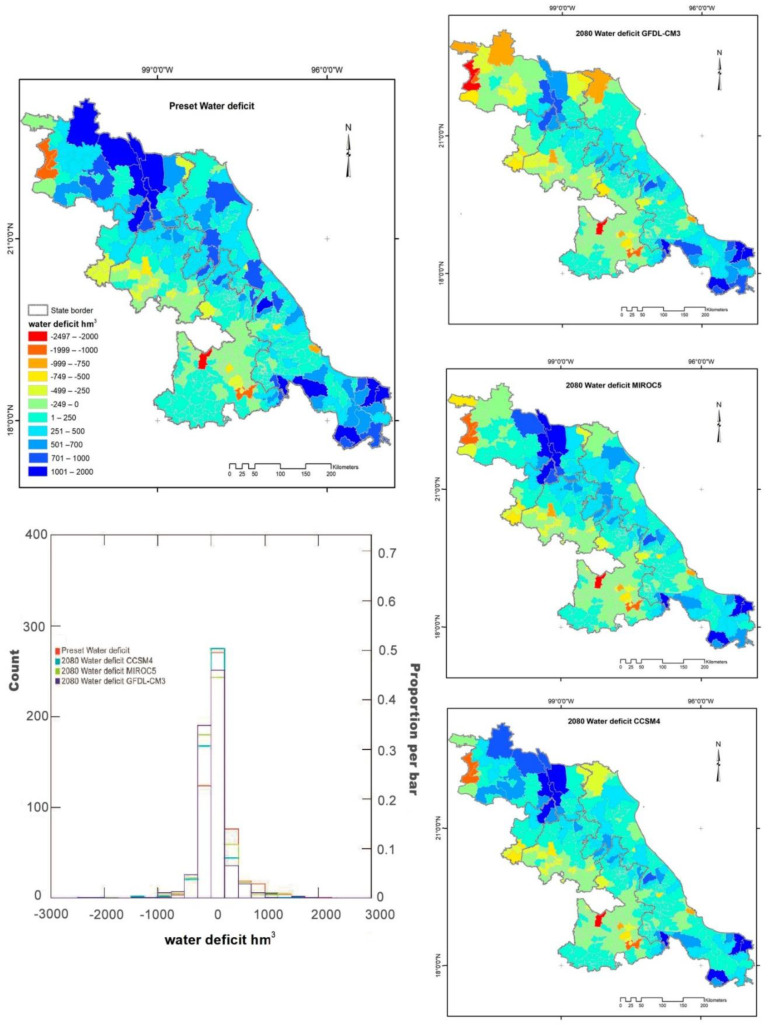
Geographical distribution of the water deficit: for the present and by 2080, considering GCM-CMIP5 climate models, under one emission scenario RCP 8.5. The change histograms of each model were overlaid to show the difference between them.

The municipalities with the highest water availability were not the same as those with the greatest demand and vice versa. For example, 27% (153) of municipalities had a higher water demand than what is actually available. These are located in the western part of the study area (Figure 6), while municipalities with the highest water availability are located in the lowland and coastal areas (Figure 5). Under the RCP 8.5 scenario, the most extreme of all, for 2080 shows that water scarcity could occur in 199 municipalities under CCSM4 model, comparing to 214 municipalities under MIROC5, and the worst scenario would be 223 municipalities under GFDL-CM3, which cover 39% of the study area (Figure 7).

Figure 7 shows an increase in municipalities with water scarcity, with emphasis on those that currently have values in a range of 250–500 (hm^3^), which in future scenarios could have negative values. As shown in the histogram, the range of −250 to 0 (hm^3^) would dramatically increase. The blue color in the maps shows municipalities that did not present a water deficit, and they maintained this characteristic in the different scenarios (Figure 7).

Underground water is the main source for the domestic sector in the most populated municipalities, in contrast with surface water sources that are for crop irrigation. Additionally, the maps show that the high surface water availability areas form a corridor from north to southeast in the Sierra Madre Oriental (Figure 7).

## 4. Discussion

Water scarcity affects more than 40% of the world population [33,34]. Considering the socio-environmental effects of drought, it should be highlighted that in the study area, drought conditions could increase by 200% in the scenarios for 2080. The global trend is an increase in water shortage, mainly due to increased demand for domestic uses and irrigation [3,33]. Population growth and associated food needs are indirect drivers of water deficit, being more marked in countries with large urban zones [35]. In recent years, the cultivation of crops for cattle feed has increased, particularly in South America [36]. In Mexico, the decrease in total annual precipitation under climate change scenarios will contribute to water shortages in the near future. Water demand is evident due to the growth of cities in the central-eastern region of Mexico.

The high environmental heterogeneity of the study region generates very diverse land uses and different levels of exposure to extreme weather events and climate change. The above can partly explain the lack of spatial correspondence between availability and demand of water. This seems to be a global phenomenon, where in many cases the demand exceeds water availability [37]. The spatial models enabled us to identify sites with 100% decrease in water availability, which corresponds to 6.5% of the study area. These results could encourage decision makers to consider these sites in climate change adaptation strategies, given their high exposure. Reduction in surface water availability would also have a negative effect on infiltration [9], which in turn would affect groundwater deposits. Furthermore, it has been reported that future models of surface runoff from the watersheds of Mexico predict reductions of up to 28% [9].

The use of water footprint as a proxy for water demand enabled us to include data in the models that, in general, have not been directly recorded by Mexican government agencies. Using water footprint, it was possible to include the water used in crop agriculture (irrigated and non-irrigated), livestock farming (intensive and extensive), and water used in the entire food production process. It is important to note that we have observed that the values of concessioned water by the government [5] did not match water footprint estimates. However, water footprint could overestimate water used because of import and export of water, especially in the domestic sector. That is, we are not considering that, for example, food products imported from other regions include water. Moreover, agricultural products from the study area could be exported to other regions or countries. Despite these drawbacks, water footprint is a simple and quick estimation tool that provides an approximation of hydrological service requirements [7,22,38]. We did not include the water footprint from the industrial and energy sectors, because there were no data at the municipal level.

In Mexico, the greatest demand for water comes from the crop agricultural sector [24], which is consistent with global estimates [39]. However, contrary to what was expected for the central-eastern region, the domestic use sector was slightly higher than the crop agricultural sector, both now and in the future models. Population is concentrated in several cities such as the metropolitan area of Puebla with a population of 2.3 million and San Luis Potosí with 825,000 inhabitants, both with high water demand for domestic use. However, if livestock and forestry water demands are added to crop agricultural demand, the result is 30,958.65 hm^3^/year, which represents 2730.17 hm^3^/year more water than the water demand of the domestic sector. It is well known that rapid population growth, urbanization, and climate change are increasingly diminishing the availability and quality of water in cities [40]. This trend was identified in some developing countries [41]. This is why the challenge is to create innovative practices to improve water security and provide better services, particularly in urban areas, considering people, policies, and places [42].

This research shows the same tendency found in the Mezquital Valley, a semidesert area highly threatened by climate warming and the high growth of its industry and agriculture. Researchers have recently found high water scarcity values on surface water and groundwater in climate change scenarios [43].

The pattern of rainfall changes under climatic change scenarios and is highly uncertain [30]. There are not evident differences among RCP scenarios in the study area, but spatial variation occurs between GCMs, and it is evident. However, at the regional and local scale it was possible to identify variation mainly to a farther future. For water deficit estimations, we used the current water demand both for the present and for 2080. Although the demand for water in the future was not estimated, it is expected that it will increase as a consequence of population growth. This implies that the water deficit was underestimated, so the water shortage scenarios may be greater for the region studied. 

In all GCMs used in this study, a significant reduction in water availability was found. Similar results to this study were found in the Las Vegas Valley, where future changes in water supply and demand were evaluated using multimodel analysis CMIP3 and CMIP5; in this study, the rate of increase in water demand gradually decreased in the future as the population growth rate was forecasted to do the same. This result is maintained in 27% of the 97 CMIP5 evaluated models, those predicted that the future mean lake level dropping below the historical mean level [44]. Other research in Colorado River Basin compared multimodel CMIP5. The results showed that the trends in temperature models were relatively consistent with the observed trends, in direction and magnitude, but showed variation in precipitation. In this work, the authors recommended the use of multiple CMIP5 models to compare results [45]. CMIP5 models show significant improvements in simulations of key Pacific climate models and their teleconnections to North America [46]. The data used were processed by WorldClim, which already includes most of the data of weather stations for its original calibration in Mexico. It presents a downscaled scale at 1 km of resolution [19]. The performance of GCMs in a region is linked to their capability to simulate the historical temperature and precipitation of the region [47]. According to the evaluation of several models in Mexico, models with the finest resolution such as a MIROC5 have better capability to simulate precipitation, while for temperature, most of the models have good skill [29]. 

A recent study showed an increase in withdrawal of water during 2010–2050, principally in developing countries such as Mexico, under more pessimistic socioeconomic pathways (SSP 3) [48]. Our water demand scenario is similar to SSP 3 but with data to present, so the water deficit for 2080 is an underestimation. The results obtained in our study generate an alarm in a large part of the region. In contrast, water deficit could decrease with sustainable politics, for example, environmentally friendly technologies, low population growth, reduction in irrigation area, and improved irrigation efficiency [48].

## 5. Conclusions

The spatial distribution of hydrological services is an important element of watershed management, including public policies that focused on establishing mitigation and adaptation to climate change in the near future [49,50]. In Mexico, municipal governments are vital actors in the conservation and management of water. However, they often lack water availability and require information for good management.

- The model that predicts a greater reduction in total water availability was GFDL-CM3 (20–40%), contrasting with the CCSM4, which predicts a lower reduction (15–28%). In the RCP 4.5 scenario, water availability was reduced by between 15% and 26% by 2050 and by 14–29% by 2080, whereas for RCP 8.5, the reduction was greater; 19–26% by 2050 and 25–40% by 2080.
- The greatest demand of water was associated with domestic use (48%), followed by crop agriculture (27%), livestock agriculture (20%), and timber production (5%).
- The results of this study can support water management at the sub-watershed and municipality level. It was seen that 27% of municipalities had a higher water demand than what it is actually available, and this would increase to 39%.

In addition, we highlighted the priority sites for conservation, and we recommend the efficient use of water because of the high risk of water deficit. To achieve this goal, all stakeholders must participate in the management of water resources to avoid reducing food production and domestic water services, which would have a negative impact on human well-being.

## Figures and Tables

**Table 1:** Water footprint for crop agricultural sector using the averages of the annual production 2000 to 2012.

Products	m^3^/Ton/Year	Products	m^3^/Ton/Year
Bovine meat	13,500	Alfalfa	676
Goat meat	10,252	Oats	1788
Chicken meat	2977	Jalapeño pepper	379
Turkey meat	2977	Bean	3177
Sheep meat	16,875	Corn grain	1744
Pig meat	6559	Pastures	450
Wax	2967	Sorghum grain	1212
Eggs	4277	Red tomato	2755
Honey	1563	Green tomato	2140
		Wood	1116

## Data Availability

Data sharing not applicable. No new data were created or analyzed in this study. Data sharing is not applicable to this article.
